# Single Epididymis Metastasis 12 Years After Radical Prostatectomy: A Case Report and Literature Review

**DOI:** 10.1155/criu/7984631

**Published:** 2026-06-12

**Authors:** Antonio Fanelli, Francesco Troiano, Francesco Guzzi, Marco Finati, Anna Ricapito, Oscar Selvaggio, Ugo Falagario, Gian Maria Busetto, Luigi Cormio, Carlo Bettocchi, Giuseppe Carrieri

**Affiliations:** ^1^ Department of Urology, University of Foggia, Foggia, Italy, unifg.it

**Keywords:** epididymis, neoplasm metastasis, orchiectomy, positron-emission tomography computed tomography, prostatic neoplasms

## Abstract

**Introduction:**

Metastatic spread of prostate cancer to the epididymis is exceptionally rare. Recognition is challenging because clinical and ultrasonographic findings may mimic benign or primary paratesticular lesions.

**Case Presentation:**

We describe a 71‐year‐old man who developed an isolated epididymal metastasis 12 years after radical prostatectomy for high‐risk prostate cancer. After adjuvant treatment and later biochemical recurrence managed with salvage radiotherapy and androgen deprivation therapy, prostate‐specific antigen rose to 2.2 ng/mL despite castrate testosterone levels. Choline positron emission tomography/computed tomography identified a suspicious paratesticular lesion. Scrotal ultrasound showed a cystic mass initially suggestive of a benign epididymal lesion. Surgical exploration with hydrocelectomy and excision of the epididymal mass was performed for both diagnostic and therapeutic purposes. Histology and immunohistochemistry confirmed metastatic adenocarcinoma of prostatic origin. After surgery, prostate‐specific antigen became undetectable.

**Conclusion:**

This case underlines the need to consider epididymal metastasis during follow‐up when prostate‐specific antigen rises without typical sites of relapse and supports the role of dedicated scrotal evaluation, molecular imaging, and individualized surgical management in selected patients.

**Keynote Message**

• An unusual epididymal metastasis appeared 12 years after radical prostatectomy.
• Rising prostate‐specific antigen prompted targeted imaging and surgical exploration.
• Timely recognition allowed excision and complete biochemical response.
• Clinicians should examine the scrotum and consider dedicated imaging during biochemical recurrence.

## 1. Introduction

Prostatic neoplasms commonly metastasize to lymph nodes and bone; involvement of the epididymis is exceedingly rare. Reported routes include retrograde lymphatic or venous spread, arterial embolization, and intraluminal dissemination through the vas deferens. We report a late isolated epididymal metastasis following radical prostatectomy and multimodal therapy and contextualize it with a focused review.

## 2. Case Presentation

A 59‐year‐old man presented in 2011 with a prostate‐specific antigen (PSA) level of 9 ng/mL. Prostate biopsy demonstrated acinar adenocarcinoma, Gleason score 3 + 4 = 7, involving 23% of sampled tissue, with perineural invasion confirmed on histopathological examination. Preoperative staging, including standard cross‐sectional imaging and bone scintigraphy, showed no evidence of metastatic disease.

The patient underwent open radical prostatectomy with bilateral pelvic lymphadenectomy. Final pathology revealed acinar adenocarcinoma upgraded to Gleason score 3 + 5 = 8, with extensive perineural invasion, bilateral lobe involvement, bilateral seminal vesicle invasion, focal positive apical surgical margin, and no nodal metastases (0/20), corresponding to pT3bN0 with R1 margins.

Adjuvant radiotherapy to the prostate bed/pelvic region and postoperative bicalutamide therapy were subsequently administered. PSA became undetectable after primary treatment.

In 2019, biochemical recurrence was diagnosed after restaging investigations showed no evidence of distant metastatic disease. The patient was treated with salvage radiotherapy (70 Gy in conventional fractions, delivered using a seven‐field conformal technique) and androgen deprivation therapy with leuprorelin.

In January 2023, PSA increased to 2.2 ng/mL despite castrate testosterone levels (0.17 ng/mL). Choline positron emission tomography/computed tomography (PET/CT) did not demonstrate widespread disease but identified a suspicious paratesticular lesion. Scrotal ultrasound demonstrated a cystic lesion measuring approximately 60 × 40 mm, initially suggestive of a benign epididymal lesion.

Because the lesion appeared isolated and histological confirmation was required, surgical exploration was undertaken with both diagnostic and therapeutic intent. Hydrocelectomy and excision of the epididymal mass were performed. Histopathological examination confirmed metastatic adenocarcinoma of prostatic origin, supported by immunohistochemical analysis demonstrating NKX3.1 positivity, confirming the prostatic origin of the lesion. Postoperatively, PSA became undetectable (< 0.09 ng/mL), supporting complete biochemical response after surgery.

A multidisciplinary team subsequently recommended prostate‐specific membrane antigen positron emission tomography/computed tomography (PSMA PET/CT), which demonstrated no additional sites of disease.

## 3. Discussion

Testicular and paratesticular metastases from prostatic neoplasms are rare (approximately 0.06%–0.5% in autopsy series), and epididymal localization is rarer still. Prostate cancer most commonly metastasizes to bone and lymph nodes, whereas involvement of the epididymis is exceptionally rare [1].Historical reports describe incidental findings at orchiectomy or autopsy, whereas contemporary cases increasingly involve detection during biochemical recurrence using advanced molecular imaging.

Epididymal metastases are diagnostically challenging because they may mimic benign cysts, hydrocele, or primary paratesticular tumors on physical examination and ultrasound imaging, as occurred in the present case.Metastatic involvement of the testis and adjacent scrotal structures may clinically mimic primary testicular neoplasms, making histopathological confirmation essential [2].

Several mechanisms have been proposed to explain this unusual metastatic pattern, including retrograde venous spread, retrograde lymphatic dissemination, arterial embolization, and intraluminal extension through the vas deferens.

Our case also highlights the potential value of metastasis‐directed surgery in selected patients with apparently isolated recurrence. Complete excision of the lesion was followed by biochemical response, suggesting that the epididymal metastasis represented the dominant active site of disease at that time.

Our case reinforces the importance of careful scrotal examination and selective imaging when PSA rises without conventional sites of relapse. Findings such as scrotal enlargement, hydrocele, palpable nodules, discomfort, or asymmetry should prompt further dedicated evaluation.

The summary of previously reported cases is presented in Table 1.

**Table 1:** Reported cases of epididymal metastasis from prostate cancer.

Author (year)	Age	Interval	Site	Detection/notes
Humphrey [3]	63	NR	Epididymis	Autopsy, first description
Kovi [4]	73	NR	Epididymis	Solitary epididymal metastasis
Talbot [5]	70	5 years post‐RP	Epididymis/Cord	After prostatectomy + vasectomy
Bahnson [6]	66	4 years post‐RT	Epididymis	Incidental at hydrocele surgery
Sneiders [7]	77, 81	> 2 years post‐TURP	Epididymis	Bilateral in one case
Rizk [8]	63	NR	Epididymis	Mimicked adenomatoid tumor
Wiebe [9]	86	NR	Epididymis	Mimicked cystadenocarcinoma
Johansson and Lannes [10]	62–84	NR	Scrotal organs	Five‐case series
Serfling [11]	73	8 years post‐RP	Epididymis	PSMA PET/CT; PSA 1.1 ng/mL; surgery response
Polverari [12]	66	5 years post‐RP	Epididymis	PSMA PET/CT; solitary lesion
Santos‐Lopes [13]	69	6 years post‐RP/RT	Epididymis	PSMA PET/CT; histology confirmed

### 3.1. Literature Review

A focused narrative review of the English‐language literature, including case reports and small case series, suggests that fewer than 30 well‐documented cases of epididymal metastasis from prostate cancer have been reported to date. Early accounts include Humphrey [3] and subsequent single cases through the 1970s–1990s. Modern reports highlight detection using PSMA PET/CT and successful metastasis‐directed surgery.

## 4. Conclusion

Epididymal metastasis should be considered in the differential diagnosis of patients with biochemical recurrence after radical prostatectomy, particularly when PSA rises without typical sites of relapse. Careful scrotal examination, appropriate molecular imaging, and timely surgical management may allow individualized treatment in selected cases.

## Funding

No funding was received for this manuscript.

## Consent

Written informed consent for publication (text and images) was obtained from the patient.

## Conflicts of Interest

The authors declare no conflict of interest.

## Data Availability

Research data are not shared.
